# Introducing the Concept of Label-Prosthesis Mismatch and Its Classification

**DOI:** 10.1016/j.shj.2026.100807

**Published:** 2026-01-29

**Authors:** Jerome Soquet, Astrid Gerritje Maria van Boxtel, Tjark Ebels

**Affiliations:** a Cardiothoracic Surgery, Royal Adelaide Hospital, Adelaide, South Australia, Australia; b Department of Cardio-Thoracic Surgery, University Medical Centre Groningen, Groningen, The Netherlands

**Keywords:** Aortic valve replacement, Implantable medical device, Prosthesis-patient mismatch, Prosthetic valves

## Abstract

• All stented bioprosthetic aortic valves are mislabeled.
• Labeled diameters are oversized compared to true diameters with various severity.
• Label oversizing has been converted into the concept of label-prosthesis mismatch.
• A simple classification of label-prosthesis mismatch is described (grade 0-6).
• This study should help surgeons in their choice of prosthetic valves.
• This study should also be an incentive for the industry towards correct labeling.

## Graphical abstract

**Figure:**
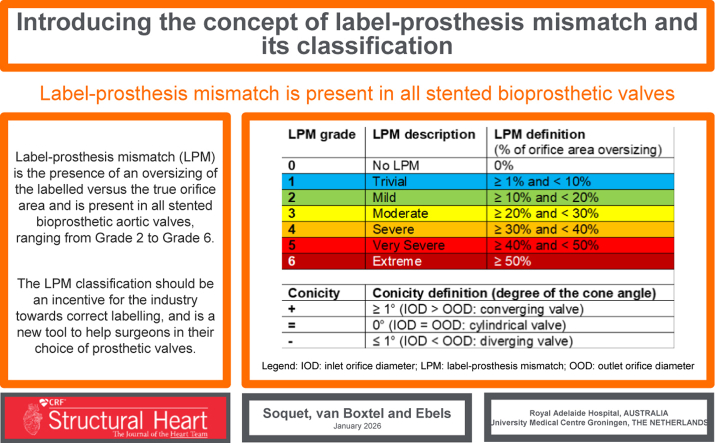

Severe and even moderate prosthesis-patient mismatches (PPMs) are associated with excess all-cause and cardiac mortality following surgical aortic valve replacement.^1^ To maximize the size of implanted valvar prostheses and minimize the risk of PPM, supra-annular implantation of selected prostheses can be performed. When the aortic annulus is too small to allow for an adequately sized prosthesis, an aortic root enlargement can be performed.^2^ In parallel, mislabeling of valvar prostheses by manufacturers has been going on for decades, and it may play a role in the implantation of prostheses that result in PPM. Based on the studies of van Boxtel et al.,^3^^,^^4^ we provide a new and simple tool to guide surgeons in their choice of the most adequate prosthetic valves according to their true size. This will be realized by introducing the concept of label-prosthesis mismatch (LPM) and its grading system.

This analysis is exclusively based on our earlier publications on eight stented bioprosthetic valves.^3^^,^^4^ The calculated orifice area from the published diameters was used as the primary criterion to define LPM and build the classification, because what physiologically matters in fine is the orifice area of the prosthesis rather than its diameter. The percentage of orifice area oversizing was calculated by dividing the orifice area derived from the labeled diameter (LD) (cm^2^) by the manufactured orifice area derived from the measured inlet orifice diameter (IOD) (cm^2^): ([(π × [LD/2]^2^)/(π × [IOD/2]^2^)]−1) × 100. LPM was defined as the presence of an oversizing of at least 1% of the orifice area derived from the LD compared to the orifice area derived from the measured (and true) IOD. Based on the severity of orifice area oversizing, a classification of LPM was built from grade 0 to grade 6 (Supplemental Table 1): grade 0 = no LPM; grade 1 = trivial LPM; grade 2 = mild LPM; grade 3 = moderate LPM; grade 4 = severe LPM; grade 5 = very severe LPM; and grade 6 = extreme LPM.

The classification of LPM was refined by adding a symbol (+, =, or −) to the LPM grade, depending on the cone angle (degrees) of the bioprosthetic valve that was deducted from the outlet orifice diameter (OOD) and the height.^4^ Converging valves (IOD > OOD) were categorized as “+,” cylindrical valves (0°, IOD = OOD) as “=,” and diverging valves (OOD > IOD) as “−.” Prostheses with positive angles (converging valves) are built with an outlet orifice that is smaller than their inlet orifice; this increases flow contraction and worsens the oversizing if present. For instance, a model that is graded 2+ is leaning toward grade 3 because its OOD is smaller than its IOD.

LPM of various degree was found in every model (grade 1 to grade 6) (Table 1). There was no grade 0 (i.e., no LPM) device. This was expected given the constant presence of label oversizing across the eight devices.^3^ Based on individual grading, the eight studied models were classified with variable severity of LPM and the type of conicity (Table 1, Supplemental Figure 1).

**Table 1:** Listed alphabetically, bioprosthetic valves and their respective grade of label-prosthesis mismatch according to orifice area oversizing and conicity

Abbreviations: LPM, label-prosthesis mismatch; NA, not applicable.

*“Mal nommer un objet, c’est ajouter au malheur de ce monde”* (To name wrongly is to add to the world’s sorrow) (Albert Camus, 1944). Efforts still need to be made by companies to label devices accurately. One goal of this report is to encourage manufacturers to do so. The other intention is to provide surgeons with a simple tool to help with decision-making because of the multiplicity of labeling methods across different companies and different models. It is meant to be a temporary tool to simplify the complexity of incorrect labeling as much as it is a helpful tool in the transition to correct labeling.

Our analysis is not exhaustive. Only stented aortic bioprosthetic valves were included, and mitral bioprosthetic valves, as well as aortic and mitral mechanical valves, are expected to be part of a next manuscript. Ideally, unstudied devices would be sent for analysis to the Groningen group to continue studying LPM with a consistent method and expand the range of graded devices. We acknowledge the fact that the grading system may be arbitrary but believe it is fairly gradual, coherent, and understandable.

## Ethics Statement

Ethics approval was not required because no human or animal subjects were involved.

## Funding

The authors have no funding to report.

## Disclosure Statement

The authors report no conflict of interest.
