# Supplementary material for: Introducing the Concept of Label-Prosthesis Mismatch and Its Classification
Source: Struct Heart. 2026 Jan 29;10(5):100807. doi: 10.1016/j.shj.2026.100807 (PMC13049503; doi:10.1016/j.shj.2026.100807)
Supplement: Supplemental Table 1 and Figure 1 [file mmc1.docx]

**Supplemental material**

**Table. Classification of label-prosthesis mismatch severity and prosthetic valve conicity**

| **LPM grade** | **LPM description** | **LPM definition (percentage of orifice area oversizing)** |
| --- | --- | --- |
| **0** | No LPM | 0% |
| **1** | Trivial | ≥ 1% and < 10% |
| **2** | Mild | ≥ 10% and < 20% |
| **3** | Moderate | ≥ 20% and < 30% |
| **4** | Severe | ≥ 30% and < 40% |
| **5** | Very Severe | ≥ 40% and < 50% |
| **6** | Extreme | ≥ 50% |
|  | | |
| **Conicity** | **Conicity definition (degree of the cone angle)** | |
| **+** | ≥ 1° (IOD > OOD: converging valve) | |
| **=** | 0° (IOD = OOD: cylindrical valve) | |
| **-** | ≤ 1° (IOD < OOD: diverging valve) | |

Abbreviations: IOD: inlet orifice diameter; LPM: label-prosthesis mismatch; OOD: outlet orifice diameter

**Figure. Proportional diagrams illustrating the least (Medtronic Avalus 29 mm) and the most (Medtronic Mosaic 23 mm) oversized models among the studied bioprosthetic valves.**


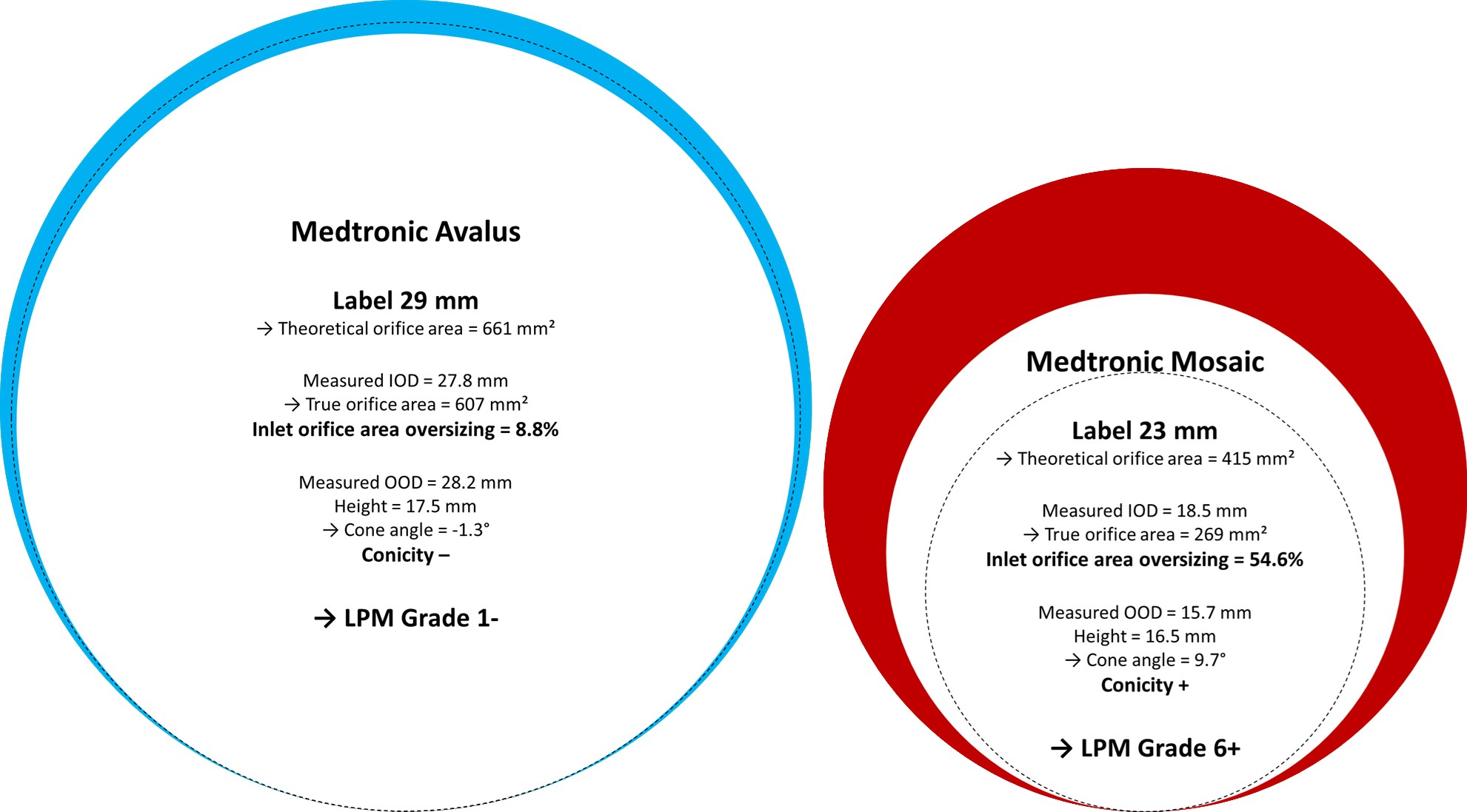


Coloured areas refer to the loss of area between the labelled inlet (external disk) and the measured inlet (white disk). The dashed black circle represents the circumference of the outlet orifice. Abbreviations: IOD: inlet orifice diameter; LPM: label-prosthesis mismatch; OOD: outlet orifice diameter
